# Racial and Ethnic Differences in the Financial Consequences of Cancer-Related Employment Disruption

**DOI:** 10.3389/fonc.2021.690454

**Published:** 2021-07-30

**Authors:** Caitlin B. Biddell, Stephanie B. Wheeler, Rebekah S.M. Angove, Kathleen D. Gallagher, Eric Anderson, Erin E. Kent, Lisa P. Spees

**Affiliations:** ^1^Department of Health Policy and Management, Gillings School of Global Public Health, University of North Carolina at Chapel Hill, Chapel Hill, NC, United States; ^2^Lineberger Comprehensive Cancer Center, University of North Carolina at Chapel Hill, Chapel Hill, NC, United States; ^3^Patient Advocate Foundation, Hampton, VA, United States

**Keywords:** financial toxicity, cancer, survivorship, productivity loss, employment

## Abstract

**Introduction:**

Cancer-related employment disruption contributes to financial toxicity and associated clinical outcomes through income loss and changes in health insurance and may not be uniformly experienced. We examined racial/ethnic differences in the financial consequences of employment disruption.

**Methods:**

We surveyed a national sample of cancer patients employed at diagnosis who had received assistance from a national nonprofit about the impact of cancer diagnosis and treatment on employment. We used logistic regression models to examine racial/ethnic differences in income loss and changes in health insurance coverage.

**Results:**

Of 619 cancer patients included, 63% identified as Non-Hispanic/Latinx (NH) White, 18% as NH Black, 9% as Hispanic/Latinx, 5% as other racial/ethnic identities, and 5% unreported. Over 83% reported taking a significant amount of time off from work during cancer diagnosis and treatment, leading to substantial income loss for 64% and changes in insurance coverage for 31%. NH Black respondents had a 10.2 percentage point (95% CI: 4.8 – 19.9) higher probability of experiencing substantial income loss compared to NH White respondents, and Hispanic or Latinx respondents had a 12.4 percentage point (95% CI: 0.3 – 24.5) higher probability compared to NH White respondents, controlling for clinical characteristics (i.e., cancer type, stage and age at diagnosis, and time since diagnosis). Similarly, NH Black respondents had a 9.3 percentage point (95% CI: -0.7 – 19.3) higher probability of experiencing changes in health insurance compared to NH White respondents, and Hispanic or Latinx respondents had a 10.0 percentage point (95% CI: -3.0 – 23.0) higher probability compared to NH White respondents.

**Discussion:**

Compared with NH White respondents, NH Black and Hispanic/Latinx respondents more commonly reported employment-related income loss and health insurance changes. Given documented racial/ethnic differences in job types, benefit generosity, and employment protections as a result of historic marginalization, policies to reduce employment disruption and its associated financial impact must be developed with a racial equity lens.

## Introduction

Almost half of over 16.9 million cancer survivors in the United States report cancer-related financial hardship, termed financial toxicity (1–3). This multidimensional construct encompasses material financial burden, altered care-seeking behaviors, and associated psychological distress stemming from medical costs, non-medical costs (e.g., transportation, childcare), and productivity loss (4, 5). Financial toxicity can have rippling effects over time, leading to medical debt, encounters with collection agencies, reductions in assets, and ultimately bankruptcy (4, 6–11). In addition, financial toxicity may cause patients to delay or forego treatment, including oral medications, as a way of coping with mounting costs (4, 12, 13). Clinically, these cumulative effects of financial toxicity are associated with worse health-related quality of life and psychological health (3, 14), higher symptom burden (15), and heightened mortality risk (16).

Over 40% of working age cancer survivors report cancer-related employment disruption, including retiring early, switching jobs, taking paid or unpaid leave, and reducing hours worked (17). Employment disruption is a significant contributor to cancer-related financial toxicity through loss of income, making it more challenging to keep up with other medical and non-medical costs, as well as loss of employer-based health insurance coverage (4, 18, 19). The effect of employment disruption on income is influenced by an individual’s access to paid resources (e.g., sick leave, short- and long-term disability insurance) during time off (20). Given that workers in higher paying jobs are more likely to have robust benefits and protections, including paid leave and employer-subsidized health insurance (21, 22), the financial consequences of employment disruption have the potential to exacerbate existing socioeconomic and racial inequities.

Prior work has documented differences by race and ethnicity in cancer-related employment disruption, with Patients of Color more commonly reporting taking extended paid and unpaid leave, stopping work altogether, and reducing work hours (17, 19, 23, 24). Additional work has shown racial disparities in the prevalence of financial toxicity (2, 9, 13, 25), but no study to date has specifically examined racial/ethnic differences in the financial consequences of employment disruption. This study aims to fill this knowledge gap using data from a survey of individuals with cancer who received assistance from a national non-profit. It is particularly important to understand the nature of financial consequences of employment disruption in this high-risk and particularly marginalized group of patients.

## Materials and Methods

### Participants and Recruitment

We used cross-sectional survey data collected by Patient Advocate Foundation (PAF), a national non-profit providing financial assistance and case management services to individuals with chronic or life-threatening illnesses. PAF administered the Impact of Disease Diagnosis on Employment survey electronically between October 2019 – November 2019 to a nationwide sample of participants who had received case management services or financial assistance from PAF between January 2018 and September 2019. This study population aims to represent patients with demonstrated healthcare access and/or affordability challenges. Participants were emailed the survey if they were no longer receiving services at the time of survey administration and opted in to receiving survey communications. PAF sent two reminder emails over the course of three weeks. Of all the email addresses sent a survey, 26% (N=3,352) completed the electronic survey. As there was no way to confirm the validity of all email addresses, it is possible that the denominator included people with invalid email addresses, thus contributing to the lower response proportion.

From this broader sample, we used survey responses to limit the analytic sample to participants who were employed (either full- or part-time) at diagnosis and self-reported a prior stage I-IV cancer diagnosis of any type (N=691). We excluded participants who were missing data for either of the two primary outcomes or for predictor variables included in the multivariable analysis with less than 10 missing responses (10.4%, 72/691). Excluded participants did not differ from the final analytic sample, with the exception of being more likely to have unknown race/ethnicity, cancer site, education, and insurance at diagnosis (**Supplemental Table S1**). The University of North Carolina Institutional Review Board deemed this secondary analysis as non-human subjects research.

### Measurement of Financial Consequences of Employment Disruption

We operationalized our primary endpoint, financial consequences of employment disruption due to treatment, as the impact of cancer-related employment disruption on (1) household income and (2) health insurance coverage. We assessed the impact of employment disruption on household income by asking participants, “To what extent has this work disruption due to treatment negatively impacted your income?” Response options included “A great deal,” “A lot,” “A moderate amount,” “A little,” or “None at all”. For analytic purposes, we collapsed response options to compare participants who reported “A great deal” or “A lot” of income loss to those who reported “A moderate amount” or less. We also asked participants to share the estimated amount of income loss monthly and the impact of this loss on household income and report these findings descriptively.

We assessed the impact of employment disruption on health insurance coverage by asking participants, “Did the change to your employment status impact your insurance coverage?” Response options included “Yes, I lost my insurance and am still uninsured,” “Yes, I lost my insurance but eventually obtained insurance coverage again,” “No,” “Not sure/don’t know.” We compared all participants whose insurance coverage was affected (whether or not they obtained coverage again) to participants who did not lose coverage or were not sure. Among those participants who lost insurance and eventually obtained new coverage, we descriptively report on the type of new coverage obtained and how the cost and coverage of this new plan compared to their plan prior to experiencing employment disruption.

### Measurement of Resource Use

Among participants who reported taking what they considered to be a significant amount of time off work during treatment, we asked about the types of resources used during absences from work. Participants were given the following options and could select all that applied: Family Medical Leave Act (FMLA), Short Term Disability Insurance (STDI), Long Term Disability Insurance (LTDI), Sick leave, Paid time off/Vacation, Unpaid Leave, Other.

To account for trends in participant response options, we assigned participants to one of three groups on the basis of their self-reported resource use: Paid Leave Only, Paid and Unpaid Leave, Unpaid Leave/No Resources. Apart from FMLA, which provides individuals protected leave from work that may be unpaid or paired with paid leave, the remaining resource categories are clearly delineated as paid (STDI, LTDI, Sick Leave, PTO/Vacation) or unpaid (Unpaid Leave/No Resources). We thus categorized participants based on the distribution of their responses across all resource categories (e.g., a participant selecting Sick Leave and PTO/Vacation only would be categorized as using “Paid Leave Only”). Participants reporting using unpaid leave only or not reporting any resources were categorized as “Unpaid Leave/No Resources.”

### Measurement of Covariates

The primary covariate in this analysis is self-reported race/ethnicity. We collapsed race and ethnicity into the following categories based on how the data were collected: Non-Hispanic or Latinx (NH) White, NH Black, Hispanic or Latinx, Other, and Not reported. Due to small sample sizes, the “Other” category includes participants self-identifying as Asian (n=17), American Indian/Alaskan Native (n<10), Middle Eastern (n<10), Native Hawaiian/Other Pacific Islander (n<10), Caribbean Islander (n<10), and mixed race (n<10). Counts less than 10 are suppressed for confidentiality.

Other covariates included self-reported clinical, socioeconomic, and demographic characteristics hypothesized to be associated with the financial consequences of employment disruption. Clinical characteristics (age at diagnosis, time since first diagnosis, cancer site, cancer stage) were hypothesized to influence functional limitations impacting ability to work. Socioeconomic characteristics (full *vs.* part-time employment, education, health insurance status at diagnosis) were hypothesized to influence employment type/demands influencing available accommodations and benefits, and demographic characteristics (gender, marital status) were hypothesized to influence social and financial supports and expectations.

### Analytic Methods

We first assessed differences in sociodemographic characteristics by race/ethnicity, comparing percentage differences between each racial/ethnic group to NH White participants, as they comprised the majority of our sample. We then used logistic regression models to assess unadjusted and adjusted differences in our primary outcomes, impact of employment disruption on household income and health insurance, by race/ethnicity. In adjusted analyses, we first controlled for clinical characteristics only according to the National Academy of Medicine definition of racial/ethnic disparities (26). We then added in sociodemographic characteristics to assess the extent to which socioeconomic status may mediate these disparities. In the multivariable regression results, average marginal effects for each covariate can be interpreted as the average difference in the predicted probability of each outcome, holding all other covariates constant, across all observations in the analytic sample (27). Standard errors and confidence intervals (CIs) for the marginal effects were estimated by applying the Delta method using the “margins” command in STATA 16.1 (StataCorp, College Station, TX) (28). No collinearity in the final models was detected using a variance inflation factor threshold of five.

In a secondary analysis, we assessed the association of resource use with financial consequences of employment disruption using logistic regression controlling for clinical and sociodemographic characteristics. We assessed differences in the average marginal effects and their associated confidence intervals between participants in each resource use category (Paid Leave Only, Paid and Unpaid Leave, Unpaid Leave or No Resources). We also assessed differences in the percentage of respondents reporting each resource use category by sociodemographic characteristics. All analyses were conducted in STATA 16.1 (StataCorp, College Station, TX).

## Results

### Participant Characteristics

Of the 619 participants included in the analytic sample, 63% were categorized as NH White, 18% as NH Black, 9% as Hispanic or Latinx, 5% as Other, and 5% as not reported. The majority of the sample was female (82%), employed full-time (*vs.* part-time) at diagnosis (83%), privately insured at diagnosis (71%), diagnosed between the ages of 41 and 60 years (59%), and diagnosed with a solid tumor cancer (77%). Compared to NH White participants, NH Black participants in this sample were more likely to be diagnosed at a younger age and to be single at diagnosis (**Table 1**).

**Table 1:** Descriptive statistics from a sample of employed patients with cancer who received assistance from a national non-profit, stratified by self-reported race/ethnicity (Oct – Nov 2019).

	Self-reported Race/Ethnicity				
	Non-Hispanic White	Non-Hispanic Black	Hispanic/Latino	Other^1^	Not reported
**N=619**	**392**	**110**	**56**	**33**	**28**
**Gender**					
Female	323 (82.4%)	94 (85.5%)	45 (80.4%)	25 (75.8%)	23 (82.1%)
Male	69 (17.6%)	16 (14.5%)	11 (19.6%)	8 (24.2%)	5 (17.9%)
**Marital status**					
Married or living with partner	182 (46.4%)	25 (22.7%)	27 (48.2%)	17 (51.5%)	11 (39.3%)
Single	93 (23.7%)	59 (53.6%)	18 (32.1%)	11 (33.3%)	10 (35.7%)
Divorced, widowed, or separated	117 (29.8%)	26 (23.6%)	11 (19.6%)	5 (15.2%)	7 (25.0%)
**Full *vs* part-time employment**					
Part-time	77 (19.6%)	14 (12.7%)	6 (10.7%)	2 (6.1%)	7 (25.0%)
Full-time	315 (80.4%)	96 (87.3%)	50 (89.3%)	31 (93.9%)	21 (75.0%)
**Educational Attainment**					
Two year college degree or less	220 (56.1%)	70 (63.6%)	37 (66.1%)	15 (45.5%)	15 (53.6%)
College degree (BA/BS) or more	153 (39.0%)	34 (30.9%)	19 (33.9%)	14 (42.4%)	12 (42.9%)
Other or not reported	19 (4.8%)	6 (5.5%)	0 (0.0%)	4 (12.1%)	1 (3.6%)
**Insurance coverage at the time of diagnosis**					
Private	270 (68.9%)	81 (73.6%)	44 (78.6%)	24 (72.7%)	19 (67.9%)
Public (Medicare, Medicaid, Military)	72 (18.4%)	15 (13.6%)	4 (7.1%)	8 (24.2%)	5 (17.9%)
Uninsured	28 (7.1%)	11 (10.0%)	5 (8.9%)	1 (3.0%)	2 (7.1%)
Other or not reported	22 (5.6%)	3 (2.7%)	3 (5.4%)	0 (0.0%)	2 (7.1%)
**Age at diagnosis**					
19-40 years old	57 (14.5%)	28 (25.5%)	12 (21.4%)	7 (21.2%)	4 (14.3%)
41-60 years old	222 (56.6%)	73 (66.4%)	31 (55.4%)	22 (66.7%)	18 (64.3%)
61 years or older	113 (28.8%)	9 (8.2%)	13 (23.2%)	4 (12.1%)	6 (21.4%)
**Time since first diagnosis**					
Within the last 12 months	49 (12.5%)	21 (19.1%)	8 (14.3%)	9 (27.3%)	4 (14.3%)
1 to 4 years ago	192 (49.0%)	56 (50.9%)	32 (57.1%)	15 (45.5%)	12 (42.9%)
5 or more years ago	151 (38.5%)	33 (30.0%)	16 (28.6%)	9 (27.3%)	12 (42.9%)
**Cancer Stage**					
Stage 1 or 2	146 (37.2%)	46 (41.8%)	17 (30.4%)	10 (30.3%)	7 (25.0%)
Stage 3 or 4	163 (41.6%)	46 (41.8%)	28 (50.0%)	17 (51.5%)	16 (57.1%)
Unknown	83 (21.2%)	18 (16.4%)	11 (19.6%)	6 (18.2%)	5 (17.9%)
**Cancer Site**					
Solid tumor^2^	294 (75.0%)	90 (81.8%)	46 (82.1%)	25 (75.8%)	21 (75.0%)
Blood^3^	70 (17.9%)	16 (14.5%)	5 (8.9%)	4 (12.1%)	5 (17.9%)
Not reported	28 (7.1%)	4 (3.6%)	5 (8.9%)	4 (12.1%)	2 (7.1%)

^1^Other includes Asian (n=17), American Indian/Alaskan Native (n<10), Middle Eastern (n<10), Native Hawaiian/Other Pacific Islander (n<10), Caribbean Islander (n<10), and mixed race (n<10). Counts less than 10 suppressed for confidentiality.

^2^Solid tumor cancers include breast (n=366), prostate (n=27), colorectal (n=22), gynecologic (n=12), lung (n=12), head and neck (n<10), bone (n<10), bladder (n<10), gastrointestinal (n<10), liver (n<10), endocrine (n<10), sarcoma (n<10), skin (n<10), thyroid (n<10). Counts less than 10 suppressed for confidentiality.

^3^Blood cancers include myeloma (n=74), Non-Hodgkin’s or Hodgkin lymphoma (n=15), leukemia (n=11).

### Financial Consequences of Employment Disruption

Most of the sample (83%) reported having to take what they considered to be a substantial amount of time off work during cancer diagnosis and treatment. Over 64% of the sample reported that their income had been impacted substantially (“a great deal” or “a lot”) as a result of cancer-related employment disruption. When asked to estimate the specific amount of income loss monthly, 50% of the sample estimated that their lost income was greater than $750 per month, and an additional 14% estimated lost income between $501 and $750 per month. Over 71% of the sample indicated that this loss of income had a substantial impact on their household income.

Almost one third (31%) of the sample reported that their cancer-related employment disruption impacted their insurance coverage; the majority of these participants obtained insurance coverage again (88%; 168/192). Of those who obtained insurance coverage again, however, 55% reported that this coverage was more expensive and 38% reported that it covered fewer services (*versus* 13% reporting that it covered more, 38% reported that it covered roughly the same amount, and 11% unsure or missing). Almost 40% of those who obtained coverage again reported switching to Medicare; 18% obtained coverage through the health insurance exchange, 18% regained coverage through an employer, and 18% enrolled in Medicaid. The remaining 6% were not sure what type of health insurance they obtained or did not respond.

In unadjusted analysis, compared to NH White respondents, income loss was more commonly reported by NH Black (60% *vs.* 75%) and Hispanic/Latinx (60% *vs.* 75%) respondents. Similar trends were observed for the impact of employment disruption on health insurance coverage when comparing NH White respondents to NH Black (28% *vs.* 38%) and Hispanic/Latinx (28% *vs.* 38%) respondents.

### Multivariable Analysis: Impact of Employment Disruption on Household Income

**Figure 1:**
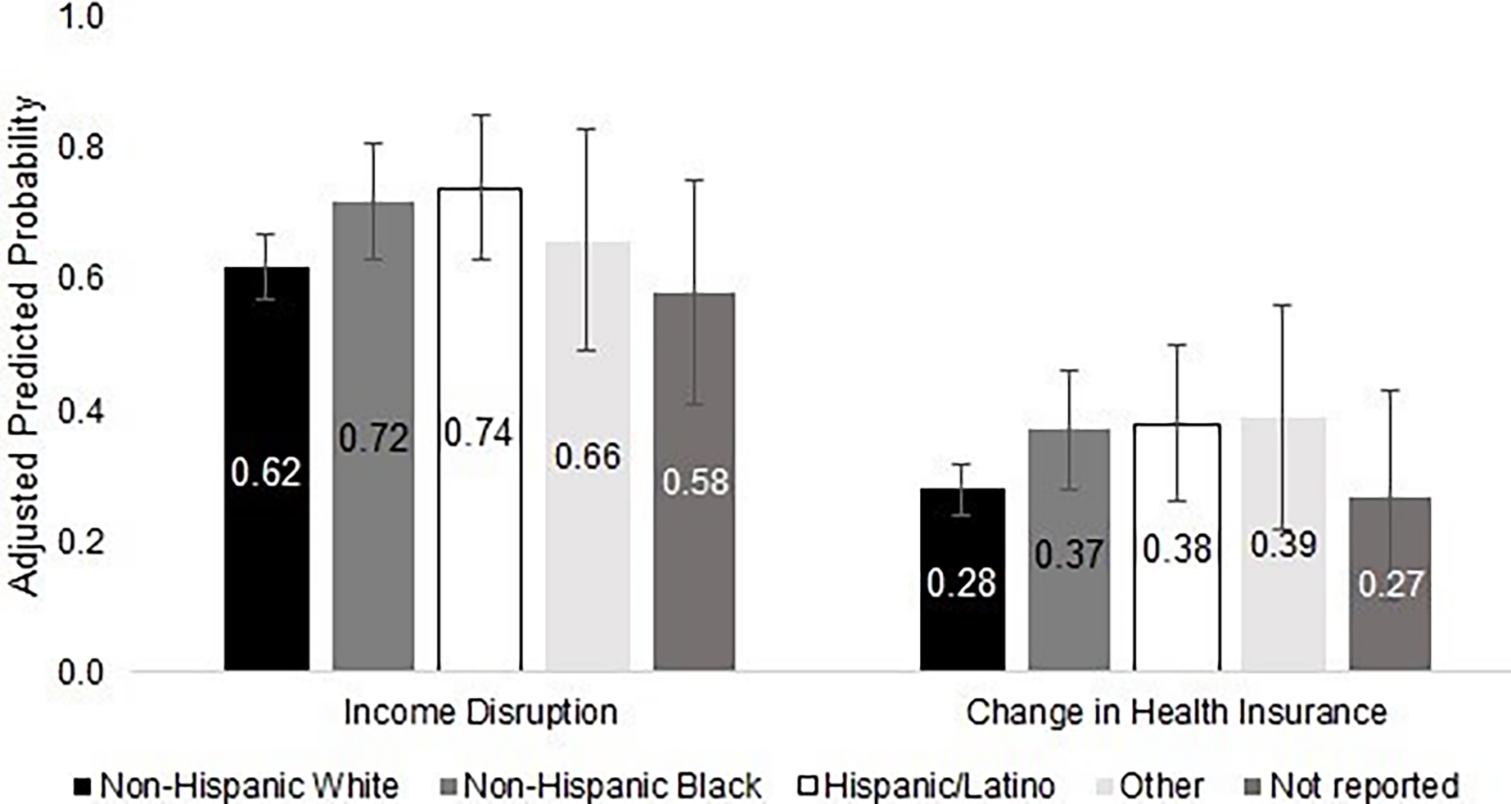
Financial consequences of employment disruption in a sample of employed patients with cancer who received assistance from a national non-profit, stratified by race/ethnicity (Oct – Nov 2019) (N = 619). **Figure 1** shows the adjusted predicted probabilities of experiencing substantial income loss and a change in health insurance following employment disruption by race/ethnicity, controlling for clinical characteristics. Adjusted percentages are reported with 95% confidence intervals from the multivariable logistic regression using Delta-method calculated standard errors.

Holding all clinical characteristics constant, NH Black respondents had a 10.2 percentage point (95% CI: 4.8 – 19.9) higher probability of experiencing substantial income loss compared to NH White respondents, and Hispanic or Latinx respondents had a 12.4 percentage point (95% CI: 0.3 – 24.5) higher probability of experiencing substantial income loss compared to NH White respondents (**Figure 1** and **Table 2**). After adding socioeconomic and demographic characteristics to the model, the difference between NH Black and NH White respondents was partially attenuated, with a marginal effect of 6.9 percentage points (95% CI: -3.2 – 17.0), but the difference between Hispanic or Latinx respondents and NH White respondents remained at 12.3 percentage points (95% CI: 0.4 – 24.2). Respondents who were younger, diagnosed with a higher cancer stage, diagnosed within the past year, diagnosed with blood (*vs.* solid tumor) cancer, non-married, employed full-time, and publicly insured or uninsured were more likely to experience income disruption (**Table 2**).

**Table 2:** Multivariable associations between patient characteristics and household income loss in a sample of employed patients with cancer who received assistance from a national non-profit (Oct – Nov 2019).

VARIABLES	Income Disruption^1^			
	*Adjusted for Clinical Characteristics Only^2^*		*Adjusted for Clinical & Sociodemographic Characteristics^3^*	
	Average Marginal Effect^4^	95% Confidence Interval	Average Marginal Effect^4^	95% Confidence Interval
**Observations**	**619**		**619**	** **
**Race/Ethnicity (ref = NH White)**				
NH Black	0.102	(0.004 – 0.199)	0.069	(-0.032 – 0.170)
Hispanic/Latinx	0.124	(0.003 – 0.245)	0.123	(0.004 – 0.242)
Other	0.039	(-0.133 – 0.211)	0.045	(-0.125 – 0.214)
Not reported	-0.034	(-0.217 – 0.149)	-0.025	(-0.204 – 0.154)
***Clinical Characteristics***				
**Cancer Stage at Diagnosis (ref = Stage 1 or 2)**				
Stage 3 or 4	0.162	(0.080 – 0.244)	0.139	(0.057 – 0.221)
Unknown stage	-0.032	(-0.142 – 0.078)	-0.020	(-0.128 – 0.088)
**Cancer Site (ref = Solid tumor)**				
Blood	0.121	(0.030 – 0.212)	0.137	(0.048 – 0.225)
Not reported	0.041	(-0.103 – 0.185)	0.032	(-0.111 – 0.174)
**Age at Diagnosis (ref = 19-40 years old)**				
41 - 60 years	-0.005	(-0.103 – 0.094)	0.003	(-0.097 – 0.103)
61 years or older	-0.159	(-0.281 – -0.036)	-0.147	(-0.274 – -0.020)
**Time Since Diagnosis (ref = < 1 year ago)**				
1 to 4 years ago	-0.122	(-0.222 – -0.022)	-0.104	(-0.206 – -0.001)
5 or more years ago	-0.188	(-0.294 – -0.082)	-0.163	(-0.273 – -0.053)
***Sociodemographic Characteristics***				
**Gender (ref = Female)**				
Male			-0.042	(-0.147 – 0.064)
**Marital Status (ref = Married, Living with Partner)**				
Single			0.091	(0.001 – 0.182)
Divorced, Widowed, or separated			0.098	(0.007 – 0.190)
**Employment Status at Diagnosis (ref = Part-time)**				
Full-time			0.115	(0.004 – 0.225)
**Educational Attainment (ref = 2 year degree or less)**				
College degree (BA/BS) or more			-0.061	(-0.137 – 0.015)
Other or not reported			-0.056	(-0.230 – 0.119)
**Insurance Status at Diagnosis (ref = Private Insurance)**				
Public (Medicare, Medicaid, Military)			0.107	(0.005 – 0.210)
Uninsured			0.202	(0.072 – 0.332)
Other or not reported			-0.026	(-0.199 – 0.147)

^1^To what extent has this work disruption due to treatment negatively impacted your income? A great deal/a lot vs. A moderate amount/a little/none at all (referent).

^2^The first column includes results from a multivariable model controlling for clinical characteristics only, specifically cancer site, stage and age at diagnosis, and time since diagnosis.

^3^The second column includes results from a multivariable model additionally controlling for sociodemographic characteristics, specifically gender, marital status, employment status at diagnosis, educational attainment, and insurance status at diagnosis.

^4^Multivariable logistic regression used to estimate average marginal effects (95% confidence intervals reported in parentheses). Average marginal effects represent the average difference in the predicted probability of experiencing income disruption, or a change in health insurance, holding all other covariates constant, across all observations in the analytic sample.

### Multivariable Analysis: Impact of Employment Disruption on Health Insurance Coverage

Controlling for all clinical characteristics, NH Black respondents had a 9.3 percentage point (95% CI: -0.7 – 19.3) higher probability of experiencing changes in health insurance compared to NH White respondents, and Hispanic or Latinx respondents had a 10.0 percentage point (95% CI: -3.0 – 23.0) higher probability compared to NH White respondents (**Figure 1** and **Table 3**). When additional demographic and socioeconomic characteristics were added to the model, the observed racial/ethnic differences were further attenuated to 7.0 percentage points (95% CI: -2.5 – 16.6) and 5.0 percentage points (95% CI: -6.9 – 16.9) for NH Black and Hispanic or Latinx respondents, respectively. Respondents who were non-married, employed full-time, and privately insured were more likely to experience a change in health insurance. Additionally, respondents diagnosed with cancer at a higher stage, blood cancer (*vs.* solid tumor), and those diagnosed more than one year prior to the survey were more likely to have a change in health insurance. Respondents age 61 years or older at diagnosis (*vs.* 19-40 years) were less likely to experience a change in health insurance (**Table 3**).

**Table 3:** Multivariable associations between patient characteristics and employment-related changes in health insurance coverage in a sample of employed patients with cancer who received assistance from a national non-profit (Oct – Nov 2019).

VARIABLES	Change in Health Insurance^1^			
	*Clinical Characteristics Only^2^*		*Clinical & Sociodemographic Characteristics^3^*	
	Average Marginal Effect^4^	95% Confidence Interval	Average Marginal Effect^4^	95% Confidence Interval
**Observations**	**619**		**619**	** **
**Race/Ethnicity (ref = NH White)**				
NH Black	0.093	(-0.007 – 0.193)	0.070	(-0.025 – 0.166)
Hispanic/Latinx	0.100	(-0.030 – 0.230)	0.050	(-0.069 – 0.169)
Other	0.108	(-0.062 – 0.278)	0.131	(-0.035 – 0.297)
Not reported	-0.009	(-0.170 – 0.153)	0.010	(-0.153 – 0.173)
***Clinical Characteristics***				
**Cancer Stage at Diagnosis (ref = Stage 1 or 2)**				
Stage 3 or 4	0.12	(0.040 – 0.200)	0.103	(0.026 – 0.180)
Unknown stage	0.056	(-0.044 – 0.155)	0.044	(-0.053 – 0.142)
**Cancer Site (ref = Solid tumor)**				
Blood	0.195	(0.084 – 0.306)	0.179	(0.072 – 0.287)
Not reported	0.094	(-0.052 – 0.240)	0.059	(-0.077 – 0.196)
**Age at Diagnosis (ref = 19-40 years old)**				
41 – 60 years	-0.008	(-0.108 – 0.092)	-0.051	(-0.149 – 0.046)
61 years or older	-0.153	(-0.265 – -0.041)	-0.132	(-0.247 – -0.016)
**Time Since Diagnosis (ref = < 1 year ago)**				
1 to 4 years ago	0.147	(0.052 – 0.242)	0.146	(0.051 – 0.241)
5 or more years ago	0.139	(0.038 – 0.240)	0.116	(0.017 – 0.215)
***Sociodemographic Characteristics***				
**Gender (ref = Female)**				
Male			0.101	(-0.005 – 0.206)
**Marital Status (ref = Married, Living with Partner)**				
Single			0.088	(0.005 – 0.171)
Divorced, Widowed, or separated			0.123	(0.036 – 0.211)
**Employment Status at Diagnosis (ref = Part-time)**				
Full-time			0.141	(0.039 – 0.243)
**Educational Attainment (ref = 2 year degree or less)**				
College degree (BA/BS) or more			-0.068	(-0.140 – 0.003)
Other or not reported			-0.23	(-0.357 – -0.102)
**Insurance Status at Diagnosis (ref = Private Insurance)**				
Public (Medicare, Medicaid, Military)			-0.233	(-0.320 – -0.145)
Uninsured			-0.176	(-0.288 – -0.064)
Other or not reported			-0.031	(-0.208 – 0.146)

^1^Did the change to your employment status impact your insurance coverage? Yes vs. No/Not sure (referent).

^2^The first column includes results from a multivariable model controlling for clinical characteristics only, specifically cancer site, stage and age at diagnosis, and time since diagnosis.

^3^The second column includes results from a multivariable model additionally controlling for sociodemographic characteristics, specifically gender, marital status, employment status at diagnosis, educational attainment, and insurance status at diagnosis.

^4^Multivariable logistic regression used to estimate average marginal effects (95% confidence intervals reported in parentheses). Average marginal effects represent the average difference in the predicted probability of experiencing income disruption, or a change in health insurance, holding all other covariates constant, across all observations in the analytic sample.

### Employment Leave Resource Use

**Figure 2:**
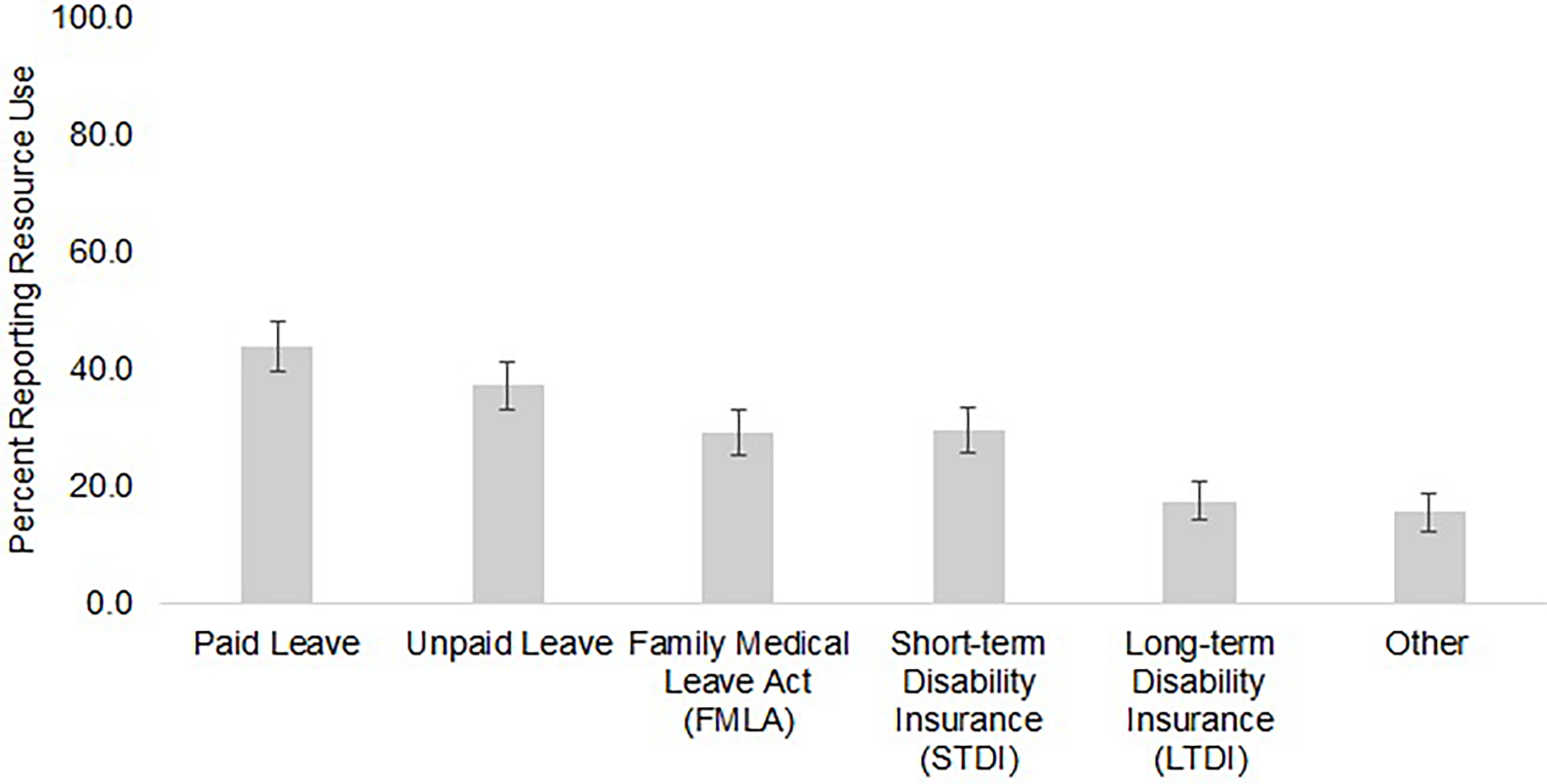
Resource use among participants who reported taking a significant amount of time off work in a sample of employed patients with cancer who received assistance from a national non-profit (Oct – Nov 2019) (N = 510). **Figure 2** shows the percentage of participants reporting taking a significant amount of time off work who reported using each type of employment leave.

**Figure 3:**
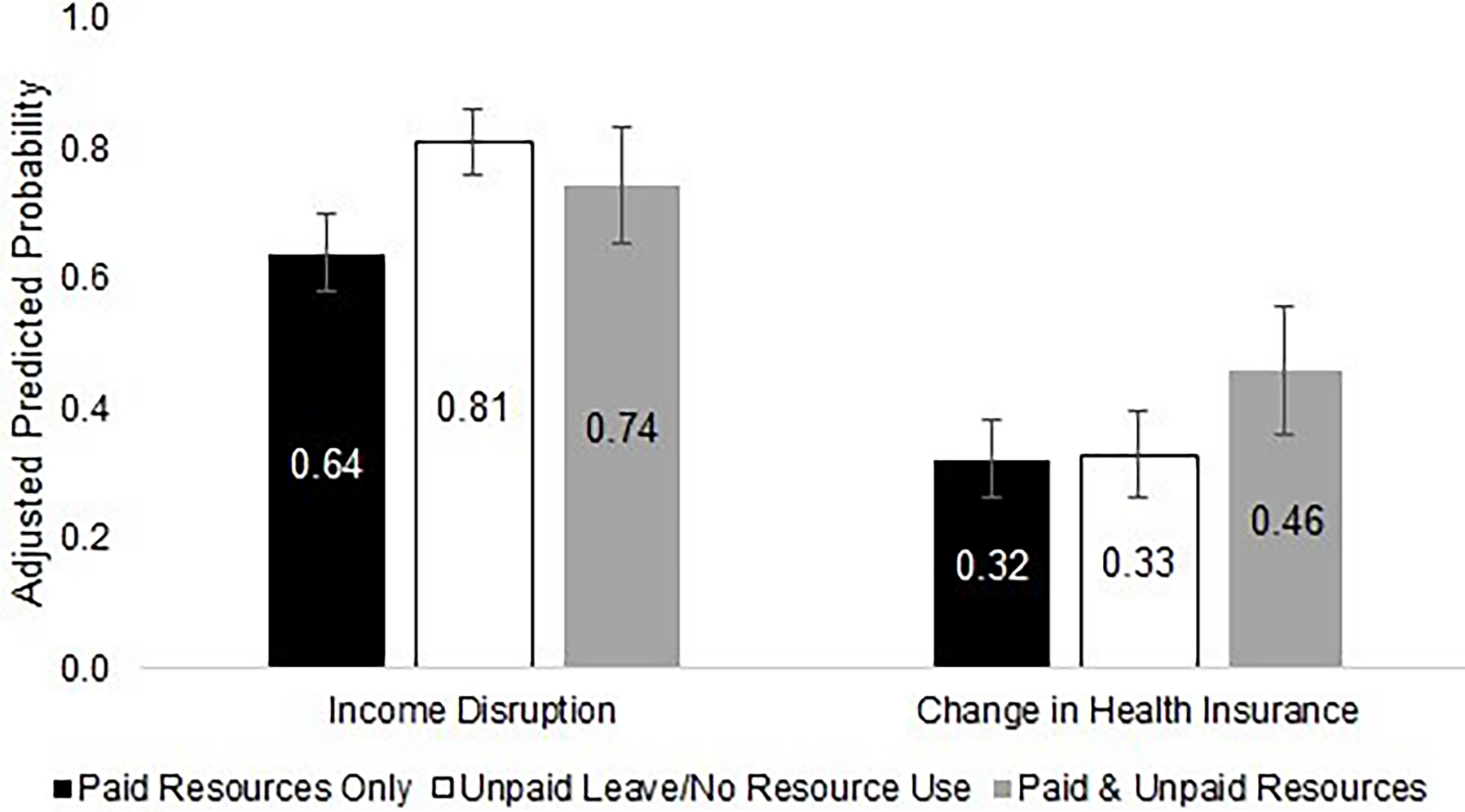
Financial consequences of employment disruption by resource use among respondents taking a significant amount of time off work in a sample of employed patients with cancer who received assistance from a national non-profit (Oct – Nov 2019) (N = 510). **Figure 3** shows the percentage of participants reporting financial consequences of employment disruption (income loss and a change in health insurance) by the types of employment leave resources used after controlling for clinical and sociodemographic characteristics. Income loss was most commonly reported among those using unpaid leave only, whereas a change in health insurance was most commonly reported among those using both paid and unpaid resources.

Among the 510 participants who reported taking what they considered to be a significant amount of time off work, **Figure 2** shows the prevalence of resource use across each resource category. Paid leave (PTO or sick leave) was reported most commonly by 44% of the sample, followed by unpaid leave reported by 37%. Almost 30% of the sample used FMLA. Short-term disability insurance was used by 30% of the sample, and only 18% reported using long-term disability insurance. After categorizing participants according to their resource use patterns, 42% used unpaid leave only or reported no resource use, 41% used paid resources only, and 17% used a mix of paid and unpaid resources (**Figure 3**). After controlling for clinical and sociodemographic characteristics, compared to participants who used paid resources only during their time off work, participants who used unpaid resources had a 17.1 percentage poin  (95% CI: 8.6 – 25.6) higher probability of reporting substantial income loss, and participants who used both paid and unpaid resources had a 14.1 percentage point (95% CI: 3.1 – 25.2) higher probability of reporting a change in health insurance (**Figure 3**).

In assessing patterns in resource use by sociodemographic characteristics, no substantial differences by race/ethnicity were observed (**Table S2**). Unsurprisingly, participants employed part-time at diagnosis more frequently used unpaid leave only compared to full-time employees. Participants with private insurance at diagnosis used paid leave only more often compared to publicly insured and uninsured participants, who were more likely to use unpaid leave only (**Table S2**).

## Discussion

Our findings are in line with prior work documenting that underrepresented patients of color are more likely than NH White patients to experience cancer-related employment disruption. However, our work provides additional detail on the financial consequences of employment disruption in a sample of patients with documented financial need, elucidating one potential mechanism underlying heightened financial toxicity in patients of color (4, 13, 29). Specifically, we identified racial/ethnic differences in the financial consequences of employment disruption, particularly income loss and changes in health insurance coverage. Even after adjusting for clinical characteristics, differences in income disruption remained between NH White, NH Black, and Hispanic or Latinx individuals. Additionally, some clinical and sociodemographic characteristics, such as stage and insurance status at diagnosis, may be acting as mediators between race/ethnicity and employment outcomes due to the impact of systemic inequities on health and socioeconomic status. As programs and policies are instituted to address patient financial and employment concerns, we must pay explicit attention to racial equity to avoid exacerbating documented racial/ethnic disparities in financial toxicity (4, 13, 29). This may include developing policies to increase employment protections and expand insurance access and designing patient-centered navigation programs to overcome structural barriers to resources and employment protections (30, 31).

The extent to which cancer impacts employment disruption is both a product of clinical and treatment characteristics (influencing how often patients must attend appointments and the symptoms/side effects experienced) (23, 32, 33), as well as characteristics of the work environment (influencing the accommodations and resources available to patients) (34, 35). Furthermore, the financial consequences of employment disruption, particularly income loss, are related to an individual’s access to resources that may provide income continuity during time off from work (e.g., paid vacation or sick leave), supplemental income (e.g., short-term and long-term disability insurance), and job security and accommodations (e.g., Family Medical Leave Act, Americans with Disabilities Act). Differences by race/ethnicity in each of these domains may help to explain our findings that NH Black and Hispanic or Latinx patients with cancer were more likely than NH White patients to experience substantial income loss throughout diagnosis and treatment after controlling for clinical characteristics.

First, NH Black and Hispanic or Latinx individuals are more likely than NH White individuals to be diagnosed with advanced disease, which frequently requires more intensive and expensive treatments, and are therefore less likely to receive recommended treatments (36, 37). These documented disparities in clinical outcomes likely influence the intensity and longevity of required treatment, as well as the functional limitations associated with cancer and treatment side effects. Second, as a result of structural racism limiting the economic opportunities of People of Color in the United States, national data show that individuals identifying as Black race and those identifying as Hispanic or Latinx ethnicity are more likely than White individuals to work in service, production, and transportation occupations (38). Further, Hispanic or Latinx individuals are more likely than both White and Black individuals to work in construction and maintenance (38). These employment categories may offer less flexible schedules, hourly *versus* salaried payment arrangements, and less opportunity for remote work (23, 39, 40), all of which have been shown to be important accommodations to individuals undergoing cancer treatment (34, 35, 41). Third, access to more generous benefit policies, including disability insurance, paid time off, and employer-sponsored health insurance, is more common among individuals in higher earning jobs and more common among White workers *versus* workers of color in the United States (21, 22, 42). Differences in access to paid benefits by race and socioeconomic status have the potential to exacerbate disparities in the financial consequences of employment disruption.

The employment-related changes in health insurance observed in a third of this sample were most likely related to the loss of private employer-sponsored health insurance (ESHI) due to extended time off, early retirement, or job loss. Thus, loss of health insurance was likely accompanied by a loss of income, compounding the experience of financial toxicity. Though we observed racial/ethnic differences in health insurance changes in unadjusted analyses, these differences were attenuated by sociodemographic characteristics, particularly marital status, employment status, and insurance status – all of which are related to the availability of and reliance on ESHI. Under FMLA, employers are required to continue offering ESHI throughout an employee’s leave; however, employees may be responsible for continuing to pay their share of the premium, which would typically be deducted from their pay (43). This may be untenable for some patients taking unpaid leave with mounting medical bills. Further, if a patient must leave work altogether, the Consolidated Omnibus Reconciliation Act (COBRA) allows most employees to retain their ESHI coverage but requires them to pay the entire premium costs previously subsidized by the employer (44). Again, this additional cost may preclude patients from taking advantage of this protection.

These results have important implications for the development of programs and policies intending to equitably intervene on financial toxicity, particularly those focusing on the financial challenges caused by employment disruption. Oncology financial navigation, in which trained navigators assist patients with financial, insurance, and employment concerns throughout treatment, is one evidence-based approach to address systemic barriers to financial and employment resources (45–49). Given that challenges associated with income loss and changes in health insurance may develop over time, this analysis underscores the changing financial needs of patients over the continuum of their cancer treatment and care. This is in line with prior longitudinal work documenting the experience of financial toxicity over time (11, 41, 50, 51), though more work in this area is needed (52). As health systems, oncology practices, and non-profit organizations increasingly implement processes and programs to identify and address patient financial concerns (45–48), it is critical to routinely check-in with patients to assess changes in needs and ongoing eligibility for different assistance mechanisms.

Furthermore, financial navigation is most effective when targeted to patients at greatest risk of financial toxicity (47). Though our analysis was primarily focused on racial/ethnic differences in the financial consequences of employment disruption, the additional sociodemographic characteristics (e.g., marital status, insurance status, age, gender, education, employment status/type) associated with both income loss and changes in health insurance in our multivariable analyses were in line with those documented in the literature on cancer-related employment disruption to date (17, 53–56). Understanding the clinical and sociodemographic characteristics associated with employment disruption and financial hardship is important for ensuring that initiatives to ameliorate financial hardship are appropriately targeted (46, 47, 57, 58).

In conjunction with programmatic interventions, policies affording workers legal protections and disability resources are critically important to ensuring job retention throughout cancer treatment and into survivorship. Only 29% of respondents in our sample who took a significant amount of time off work reported using FMLA. FMLA offers up to 12 weeks of unpaid time off with job security for individuals working at a firm with more than 50 employees who meet specific criteria for hours worked and tenure (42, 43). Additionally, the Americans with Disabilities Act (ADA) requires employers to grant requests for reasonable accommodations to employees with specified conditions, including cancer. However, the ADA does not apply to firms with 15 or fewer employees, and the employer does not have to provide an accommodation if doing so would be an undue hardship, which is largely up to the employer’s discretion (42, 59). As employees are increasingly hired in alternative contractual arrangements (60), attention must be paid to ensuring workers have equitable access to such legal protections (42). Furthermore, ensuring all patients are aware of these legal protections and have the skills and resources necessary to navigate these conversations with employers is a critical area of ongoing research to promote equity in employment outcomes (41, 61).

These findings must be viewed in the context of several limitations. The sample surveyed represents a financially vulnerable population who sought supportive services from a national non-profit; thus, conclusions drawn are not generalizable to the full US population of employed patients with cancer. The low survey response proportion also introduces the potential for selection bias if participants were more likely to respond if they had experienced extreme financial toxicity or employment disruption. This further reduces the generalizability of these study findings. As a result, it is likely that the prevalence of employment disruption, income loss, and changes in health insurance are higher in this population of patients with demonstrated financial need as compared to the broader population. However, we do not have reason to believe that this selection bias would influence the associations of patient characteristics with financial toxicity. The directional associations observed in our multivariable analyses are largely in concordance with a recent analysis of employment disruption among cancer survivors using nationally representative Medical Expenditure Panel Survey data (17). Additionally, it is critically important to understand the nature of financial needs in particularly marginalized, low-income individuals, such as those included in our sample. Future research should further investigate racial/ethnic differences in financial consequences of employment disruption in a nationally representative sample.

Another limitation is the use of self-reported measures for employment outcomes, which had not been validated in this population. Though most prior studies investigating this issue have relied on self-report (17, 19), there is a need for the validation of questionnaires and measures across diverse patient populations. Additionally, respondents’ self-identified race and ethnicity were collapsed for analysis into four mutually exclusive categories due to sample size limitations. Therefore, we did not have enough data to draw meaningful conclusions about racial/ethnic differences in employment disruption between groups other than NH White, NH Black, and Hispanic or Latinx. We also did not have data on income at diagnosis, which has been shown to be associated with employment disruption in prior work (53). Instead, we used educational attainment as a proxy for baseline socioeconomic status. The inclusion of income at diagnosis in our models controlling for clinical and sociodemographic characteristics may have further attenuated the racial/ethnic differences observed due to income potentially mediating the association of race/ethnicity with the financial consequences of employment disruption. Lastly, though we included stage at initial diagnosis in our models, we did not have information on potential stage progression. Thus, the stage data included may not fully represent a respondent’s clinical context while experiencing employment disruption.

Among a national sample of patients with cancer in financial need who obtained assistance from a non-profit organization, NH Black and Hispanic or Latinx respondents were more likely than NH Whites to experience substantial income loss and changes in health insurance resulting from employment disruption. Policies and practices to address financial hardship, and specifically the financial consequences of employment disruption, must be developed with a racial equity lens to ensure that they recognize and address the systemic inequities leading to these observed differences.

## Data Availability Statement

The datasets presented in this article are not readily available because this data is owned by Patient Advocate Foundation and is subject to a Data Use Agreement. Requests to access the datasets should be directed to https://www.patientadvocate.org/.

## Author Contributions

CB: Conceptualization, Methodology, Formal analysis, Writing – Original draft preparation. SW: Conceptualization, Methodology, Supervision, Writing – Review and Editing. RA: Investigation, Conceptualization, Writing – Review and Editing. KG: Investigation, Conceptualization, Writing – Review and Editing. EA: Data Curation, Conceptualization, Writing – Review and Editing. EK: Writing – Review and Editing. LS: Conceptualization, Methodology, Supervision, Writing – Review and Editing. All authors contributed to the article and approved the submitted version.

## Funding

CB is supported by a NIH Cancer Care Quality Training Program grant, for which SW is mentor and PI, UNC-CH, Grant No. T32-CA-116339. NIH did not have any role in the study design; collection, management, analysis, and interpretation of the data; writing of the manuscript; or the decision to submit the report for publication.

## Conflict of Interest

SW has received research grants from Pfizer paid to her institution for unrelated work.

The remaining authors declare that the research was conducted in the absence of any commercial or financial relationships that could be construed as a potential conflict of interest.

## Publisher’s Note

All claims expressed in this article are solely those of the authors and do not necessarily represent those of their affiliated organizations, or those of the publisher, the editors and the reviewers. Any product that may be evaluated in this article, or claim that may be made by its manufacturer, is not guaranteed or endorsed by the publisher.
