# Supplementary material for: Racial and Ethnic Differences in the Financial Consequences of Cancer-Related Employment Disruption
Source: Front Oncol. 2021 Jul 30;11:690454. doi: 10.3389/fonc.2021.690454 (PMC8361325; doi:10.3389/fonc.2021.690454)
Supplement: Supplementary file 1 [file Table_1.docx]

**Supplemental Appendix**

**Table S1.** Comparison of included and excluded participants

|  | **Included** | **Excluded** | p-value^1^ |
| --- | --- | --- | --- |
| **N** | **619** | **72** |  |
| **Race/Ethnicity** |  |  | **0.002** |
| Non-Hispanic White | 392 (90.1%) | 43 (9.9%) |  |
| Non-Hispanic Black | 110 (91.7%) | 10 (8.3%) |  |
| Hispanic/Latino | 56 (94.9%) | 3 (5.1%) |  |
| Other | 33 (91.7%) | 3 (8.3%) |  |
| Not reported | 28 (68.3%) | 13 (31.7%) |  |
| **Cancer Stage** |  |  | 0.108 |
| Stage 1 or 2 | 226 (90.8%) | 23 (9.2%) |  |
| Stage 3 or 4 | 270 (90.9%) | 27 (9.1%) |  |
| Unknown | 123 (84.8%) | 22 (15.2%) |  |
| **Cancer Site** |  |  | **<0.001** |
| Solid tumor | 476 (76.9%) | 48 (66.7%) |  |
| Blood cancer | 100 (16.2%) | 7 (9.7%) |  |
| Not reported | 43 (7.0%) | 17 (23.6%) |  |
| **Age at diagnosis** |  |  | 0.540 |
| 40 years old or less | 108 (87.8%) | 15 (12.2%) |  |
| 41-60 years old | 366 (89.5%) | 43 (10.5%) |  |
| 61 years or older | 145 (91.8%) | 13 (8.2%) |  |
| **Time since first diagnosis** |  |  | 0.056 |
| Within the last 12 months | 91 (85.0%) | 16 (15.0%) |  |
| 1 to 4 years ago | 307 (92.5%) | 25 (7.5%) |  |
| 5 or more years ago | 221 (88.4%) | 29 (11.6%) |  |
| **Gender** |  |  | 0.505 |
| Female | 510 (90.6%) | 53 (9.4%) |  |
| Male | 109 (88.6%) | 14 (11.4%) |  |
| **Marital status** |  |  | 0.087 |
| Married or living with partner | 262 (92.3%) | 22 (7.7%) |  |
| Single | 191 (87.2%) | 28 (12.8%) |  |
| Divorced, widowed, or separated | 166 (92.7%) | 13 (7.3%) |  |
| **Full vs part-time employment** |  |  | 0.319 |
| Part-time | 106 (92.2%) | 9 (7.8%) |  |
| Full-time | 513 (89.1%) | 63 (10.9%) |  |
| **Educational Attainment** |  |  | **<0.001** |
| Two year college degree or less | 357 (96.0%) | 15 (4.0%) |  |
| College degree (BA/BS) or more | 232 (94.3%) | 14 (5.7%) |  |
| Other or not reported | 30 (41.1%) | 43 (58.9%) |  |
| **Insurance coverage at the time of diagnosis** |  |  | **0.026** |
| Private | 438 (88.8%) | 55 (11.2%) |  |
| Public (Medicare, Medicaid, Military) | 104 (95.4%) | 5 (4.6%) |  |
| Uninsured | 47 (92.2%) | 4 (7.8%) |  |
| Other or not reported | 30 (78.9%) | 8 (21.1%) |  |

^1^ p-values calculated using Chi-squared or Fisher's Exact test.

**Table S2.** Bivariate associations between resource use and participant characteristics (N=510)

|  | **Paid Resources Only** | **Unpaid Leave/No Resource Use** | **Paid and Unpaid Resources** |
| --- | --- | --- | --- |
|  | n (row %) | n (row %) | n (row %) |
| **N** | **210 (41%)** | **102 (20%)** | **198 (39%)** |
| **Race/Ethnicity** |  |  |  |
| Non-Hispanic White | 121 (38.8%) | 134 (42.9%) | 57 (18.3%) |
| Non-Hispanic Black | 46 (47.9%) | 37 (38.5%) | 13 (13.5%) |
| Hispanic/Latino | 22 (44.9%) | 21 (42.9%) | 6 (12.2%) |
| Other | 13 (43.3%) | 10 (33.3%) | 7 (23.3%) |
| Not reported | 9 (39.1%) | 10 (43.5%) | 4 (17.4%) |
| **Cancer Stage** |  |  |  |
| Stage 1 or 2 | 77 (42.5%) | 69 (38.1%) | 35 (19.3%) |
| Stage 3 or 4 | 99 (41.6%) | 96 (40.3%) | 43 (18.1%) |
| Unknown | 35 (38.5%) | 47 (51.6%) | 9 (9.9%) |
| **Cancer Site** |  |  |  |
| Solid tumor | 153 (39.9%) | 163 (42.6%) | 67 (17.5%) |
| Blood cancer | 47 (53.4%) | 29 (33.0%) | 12 (13.6%) |
| Not reported | 11 (28.2%) | 20 (51.3%) | 8 (20.5%) |
| **Age at diagnosis** |  |  |  |
| 40 years old or less | 44 (45.4%) | 33 (34.0%) | 20 (20.6%) |
| 41-60 years old | 127 (41.8%) | 117 (38.5%) | 60 (19.7%) |
| 61 years or older | 40 (36.7%) | 62 (56.9%) | 7 (6.4%) |
| **Time since first diagnosis** |  |  |  |
| Within the last 12 months | 25 (33.8%) | 38 (51.4%) | 11 (14.9%) |
| 1 to 4 years ago | 106 (40.0%) | 115 (43.4%) | 44 (16.6%) |
| 5 or more years ago | 80 (46.8%) | 59 (34.5%) | 32 (18.7%) |
| **Gender** |  |  |  |
| Female | 175 (41.5%) | 166 (39.3%) | 81 (19.2%) |
| Male | 36 (40.9%) | 46 (52.3%) | 6 (6.8%) |
| **Marital status** |  |  |  |
| Married or living with partner | 83 (39.7%) | 94 (45.0%) | 32 (15.3%) |
| Single | 70 (42.4%) | 64 (38.8%) | 31 (18.8%) |
| Divorced, widowed, or separated | 58 (42.6%) | 54 (39.7%) | 24 (17.6%) |
| **Full vs part-time employment** |  |  |  |
| Part-time | 12 (15.0%) | 61 (76.3%) | 7 (8.8%) |
| Full-time | 199 (46.3%) | 151 (35.1%) | 80 (18.6%) |
| **Educational Attainment** |  |  |  |
| Two year college degree or less | 133 (44.0%) | 124 (41.1%) | 45 (14.9%) |
| College degree (BA/BS) or more | 72 (38.7%) | 75 (40.3%) | 39 (21.0%) |
| Other or not reported | 6 (27.3%) | 13 (59.1%) | 3 (13.6%) |
| **Insurance coverage at the time of diagnosis** |  |  |  |
| Private | 178 (49.4%) | 100 (27.8%) | 82 (22.8%) |
| Public (Medicare, Medicaid, Military) | 18 (21.2%) | 63 (74.1%) | 4 (4.7%) |
| Uninsured | 9 (20.9%) | 34 (79.1%) | 0 (0.0%) |
| Other or not reported | 6 (27.3%) | 15 (68.2%) | 1 (4.5%) |
